# Surgical resection of unilateral thalamic tumors in adults: approaches and outcomes

**DOI:** 10.1186/s12883-015-0487-x

**Published:** 2015-11-07

**Authors:** Lei Cao, Chuzhong Li, Yazhuo Zhang, Songbai Gui

**Affiliations:** Department of Neurosurgery, Beijing Tiantan Hospital, Capital Medical University, 6 Tiantanxili, Dongcheng District Beijing, China; Capital Medical University, Beijing Neurosurgical Institute, 6 Tiantanxili, Dongcheng District Beijing, China

**Keywords:** Adult, Thalamic tumor, Unilateral, Surgical approach, Clinical result

## Abstract

**Background:**

The thalamic tumors were less common in adults and this study aimed to determine the clinical features, surgical approaches, and outcomes of adult thalamic tumors, which have not been well-described in the literature.

**Methods:**

We reviewed the clinical presentation, surgical approach, perioperative mortality and morbidity, and outcomes of 111 operated patients (71 males, 40 females; mean age at presentation, 33.4 ± 13.2 years) with unilateral thalamic tumor.

**Results:**

The most common clinical presentations were increased intracranial pressure (65 %) and motor deficits (40 %). Five surgical approaches were used depending on tumor location; the most common was the transparieto-occipital approach (47.7 %). According to peri- and post-operative magnetic resonance imaging findings, the tumors were totally resected in 29 cases (26.1 %), subtotally resected in 54 cases (48.6 %), and partially resected in 21 cases (18.9 %). Five patients died during the perioperative period (4.5 %, 5/111). The most common morbidity was motor deficits (21.7 %, 23/106). According to histological findings, there were 50 high-grade and 61 low-grade tumors. Median survival of patients with low- and high-grade tumors were 40 and 12 months, respectively (mean follow-up, 37.3 months). Survival was significantly longer in cases of total or subtotal resection (median, 28 months) compared to partial resection or biopsy (median, 12 months). Survival was poorer in adults than in previous reported pediatrics.

**Conclusions:**

Surgical treatment of adult thalamic tumors must be individualized according to tumor location. Low-grade tumors and total/subtotal resection seem to be predictors of better surgical outcomes. Nevertheless, the outcome of adult patients were still worse than pediatric patients.

## Background

Thalamic tumors are rare, comprising 5 % of all brain tumors, and can occur in all age groups, but are more common in children than in adults [1, 2]. In the past, the outcome of thalamic tumors was generally poor because they are deep-seated and surrounded by vital structures, such as the internal capsule and subthalamus, and the risks of postoperative morbidity and mortality were high [3, 4]. However, recent improvements in neuroimaging and surgical techniques have made surgical resection of thalamic tumors feasible in children and have reduced the morbidity and mortality rates associated with this approach [2, 5–9]. For unilateral thalamic tumors, many studies have reported that surgical resection can improve survival of patients with low morbidity and mortality, so they recommend that surgical resection should be performed in combination with adjunct therapies, such as radiotherapy and chemotherapy, based on the histological characteristics of the tumors [10–12].

Thalamic tumors have received much less attention in adults than in pediatric patients. Moreover, the clinical characteristics, operative indications, postoperative morbidity and mortality rates, prognosis, and outcome of unilateral thalamic tumors in adults may differ from those of pediatric patients. It has been reported that thalamic tumors can be resected via several different approaches, although the indications for surgery are not well-described for adults. Correspondingly, the characteristics, treatment approach, and prognosis for unilateral thalamic tumors in adults need to be better defined. To the best of our knowledge, the present series of 111 adults with unilateral thalamic tumors is the largest to be reviewed, and both indications for surgery and clinical results were examined.

## Methods

### Patient population

This retrospective medical record review was performed in accordance with tenants of the Declaration of Helsinki. All patients gave informed consent and the study protocol was approved by the Ethical Review Board of Beijing Tiantan Hospital. Operated cases involving adult patients (>18 years old) with unilateral thalamic lesions who presented to the Capital Medical University Beijing Tiantan Hospital for the first time between 2003 and 2010 were retrospectively reviewed (*n* = 111). Tumors arising from adjacent structures (basal ganglia, optic pathways, hypothalamus, ventricle, brainstem, and pineal region) and involving the thalamic region were excluded. With regret, the “only observed” patients were not included.

### Clinical and imaging data

Clinical data, including age at presentation, duration and type of symptoms, treatment received, extent of resection, symptom improvement, and status at the end of the follow-up period, were recorded. All patients underwent magnetic resonance imaging (MRI) before treatment and during the follow-up period. Special attention was given to the tumor epicenter, its extension to adjacent structures, and the extent of tumor removal. The tumor volume was calculated using the following formula: axial *coronal *sagittal/2 (referring to the largest diameters), as described by Kramm [13].

### Surgical treatment

Based on the location of the tumor in the thalamus and surrounding structures, transfrontal, transcallosal, transtemporal, subtemporal, and transparieto-occipital approaches were used. In addition, stereotactic biopsy was performed when the patients were too weak to undergo craniotomy. Neuronavigation or ultrasonography was applied intraoperatively to position the transcortical point and define the tumor margins. Intraoperative electrophysiology was used to identify sensorimotor pathways in 46 cases since 2007. High-grade tumors were treated by additional adjunctive radiotherapy or chemotherapy. The extent of resection was defined as partial (<90 %), subtotal (>90 %), or total based on both postoperative imaging (evaluated by three experienced radiologists at our institute) and intraoperative evaluations (evaluated by the operator). The quality of life of each patient before and after surgery was evaluated using the Karnofsky performance status (KPS) score.

### Histopathological analysis

Tumor diagnoses were reviewed by three experienced neuropathologists at our institute according to the WHO 2007 classification standards for central nervous system tumors [14].

### Survival analysis

Of the 111 patients of this series, 86 underwent follow-up monitoring after surgery. Correlations were made using univariate analysis according to the Kaplan–Meier method. The threshold for statistical significance was a *p*-value of ≤ 0.05.

## Results

### Clinical presentation

Between 2003 and 2010, 111 adult patients with unilateral thalamic tumors underwent surgical resection at our hospital. This group included 71 males and 40 females (ratio: 1.8:1). In 51/111 patients (45.9 %), the tumor was on the left side. The mean age at presentation was 33.4 ± 13.2 years (range, 18–64 years) and the mean duration of symptoms was 3.6 ± 6.4 months (range, 0.25–19 months). For 28/111 patients (25 %), the tumor was present for less than 1 month prior to diagnosis, for 55/111 patients (50 %) the tumor was present for 1–2 months prior to diagnosis, and for 28/111 patients (25 %) the tumor was present for more than 2 months prior to diagnosis. The symptoms reported for the present cohort are listed in Table 1. The most common presentation was increased intracranial pressure (ICP) (72/111, 65 %), which was characterized by headaches, vomiting, and papilledema. Motor deficits were also a common presentation (44/111, 40 %).

**Table 1:** Clinical features for 111 adults with unilateral thalamic tumors

Clinical features	No. of patients (%)
Increased ICP	72 (65)
Motor deficits	44 (40)
Sensory deficits	30 (27)
Visual deficits	33 (30)
Other^a^	35 (32)

^a^Other symptoms included involuntary movement, spasticity, seizures, and behavioral problems

*ICP* intracranial pressure

All patients underwent MRI before surgery. Special attention was given to the volume and location of each tumor. The mean tumor volume was 38.4 ± 28.9 cm [3] (range, 4–140 cm [3]). Tumors were also divided into two major groups based on their location in the MR images. Tumors with an epicenter in the thalamic region were in one group, while tumors arising from the junction of the thalamus and cerebral peduncle—with most of their mass being in the thalamic region—were included in the second group. In the first group (*n* = 84), the tumor epicenter was located in the anterior thalamic nuclei (*n* = 20), the lateral nuclei (*n* = 7), the medial nuclei (*n* = 18), or the pulvinar (*n* = 39). In this group, the tumors also extended in the anterior or superior direction towards the front horn of the lateral ventricle (*n* = 14), medially toward the third ventricle (*n* = 11), laterally toward the adjacent lobe or gyrus (*n* = 7), and posteriorly toward the parietal and temporal lobes (*n* = 29). In the second group (*n* = 27), the tumors originated from the thalamus and extended to the midbrain.

### Tumor resection

**Fig. 1:**
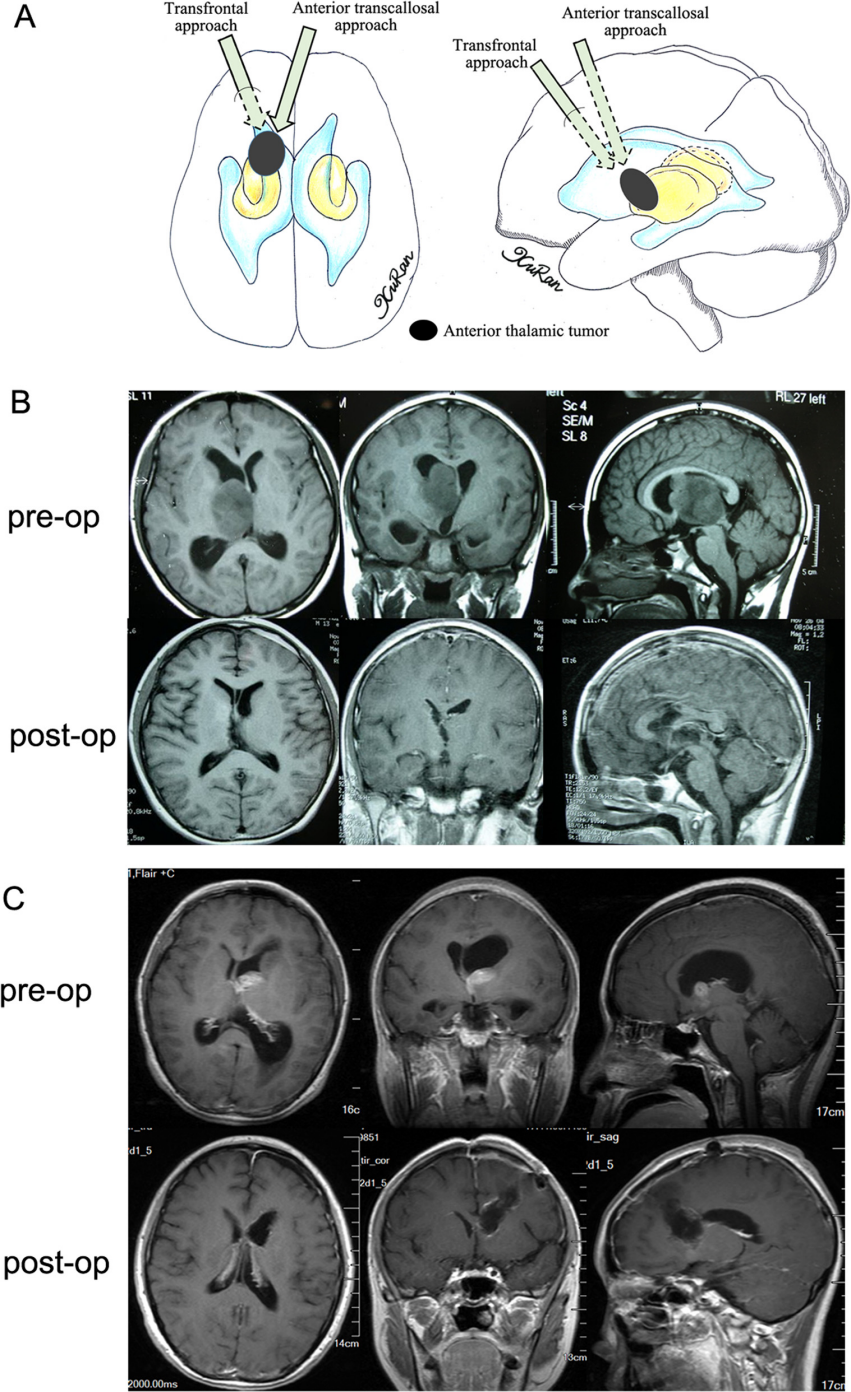
**a** Different surgical approaches to anterior thalamic tumors were showed in the diagram. **b** An anaplastic astrocytoma arising from anterior thalamus was resected totally via an anterior transcallosal approach. **c** Another anaplastic astrocytoma was resected totally via a transfrontal approach

**Fig. 2:**
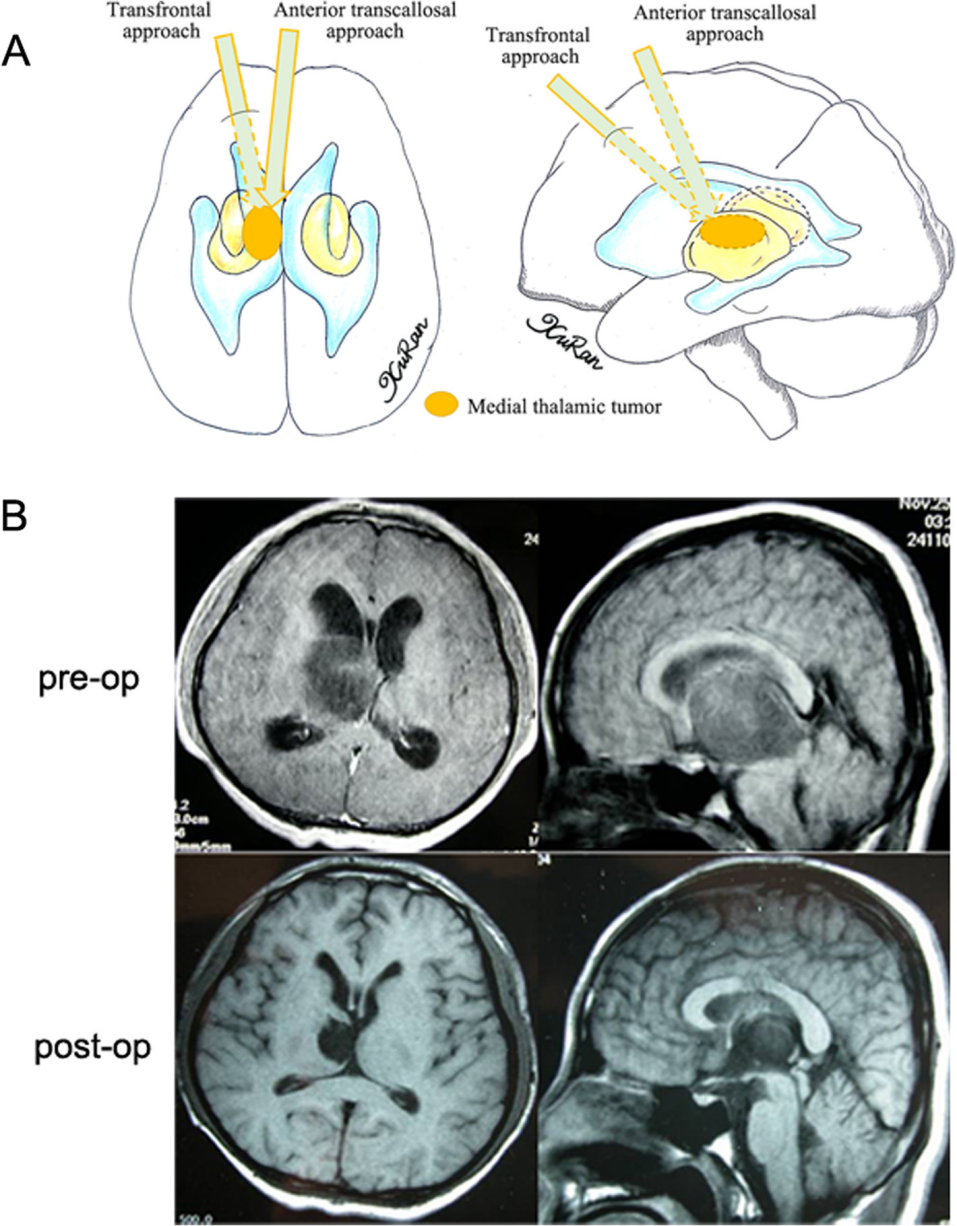
**a** Different surgical approaches to medial thalamic tumors were showed in the diagram. **b** An anaplastic astrocytoma was resected totally via an anterior transcallosal approach. The tumor extends medially to the third ventricle, as seen on the preoperative MR image. The top of the tumor is exposed after an incision into the callosum

**Fig. 3:**
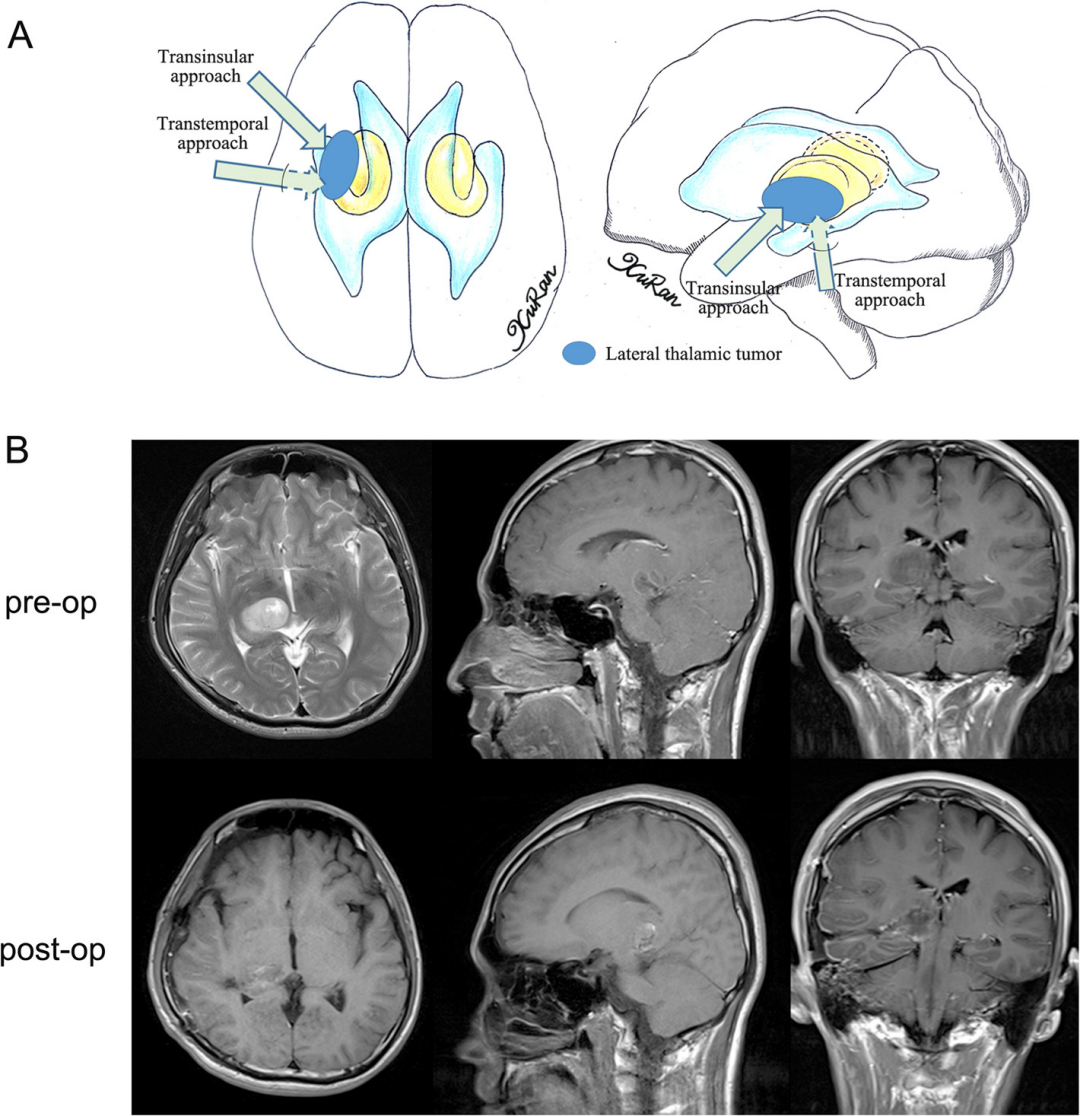
**a** Different surgical approaches to lateral thalamic tumors were showed in the diagram. **b** The anaplasia astrocytoma arose from the lateral part of the thalamus and was removed by transtemproal approach subtotally

**Fig. 4:**
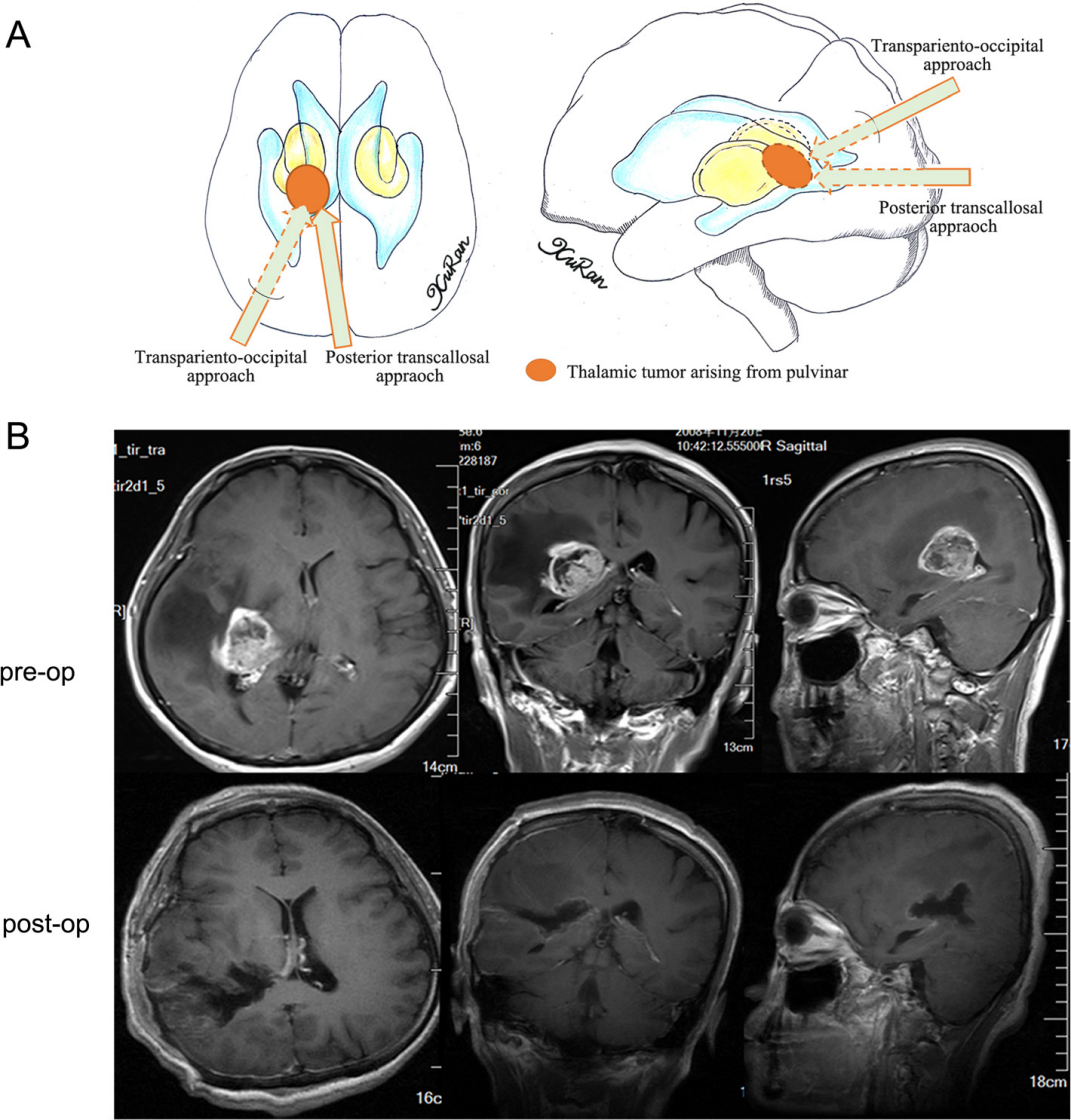
**a** Different surgical approaches to thalamic tumors arising from pulvinar were showed in the diagram. **b** A glioblastoma arose from the posterior part of the thalamus and extended posteriorly to the lateral ventricle, and was removed totally via a transparieto-occipital approach. The cortex was incised with the help of neuronavigation

According to the location of each tumor on the preoperative MR images, several surgical approaches were used. In cases where the epicenter was located in the anterior thalamus with/without anterior extension to the fontal horn of the lateral ventricle or callosum, the anterior transcallosal approach was used, provided that the tumor extended toward the lateral ventricle without causing injury to the hemisphere tissue or cortical incision. In cases where the tumor extended too far laterally, a transfrontal approach was used to reach the frontal horn of the lateral ventricle, then the ventricle floor was incised and the tumor was exposed. A transcallosal approach was used in 13 cases (12 %), while a transfrontal approach was used in 7 cases (6 %) (Fig. 1). In cases where the tumor epicenter was located in the medial thalamic region with or without extension into the third ventricle, the transcallosal approach was used (17, 15 %) (Fig. 2). A stereotactic biopsy was performed in a single case (0.9 %). In cases where the tumor epicenter was located in the lateral thalamic region with/without lateral extension to the basal ganglia, adjacent lobes, or gyrus, and when the tumor extended beneath the temporal cortex, the trans-temporal middle gyrus approach was used. The transtemporal approach was used in 7 cases (6 %) (Fig. 3). For tumors located in the pulvinar with posterior extension toward adjacent structures, a transparieto-occipital transventricular approach was used. For this approach, the cortex was incised from the border of the parieto-occipital lobes and the sulcus was dissected to reduce cortical injury and reduce the risk of postoperative seizures and hemianopsia. The transparieto-occipital approach was used in 37 cases (33 %) and stereotactic biopsy was performed in two (Fig. 4).

**Fig. 5:**
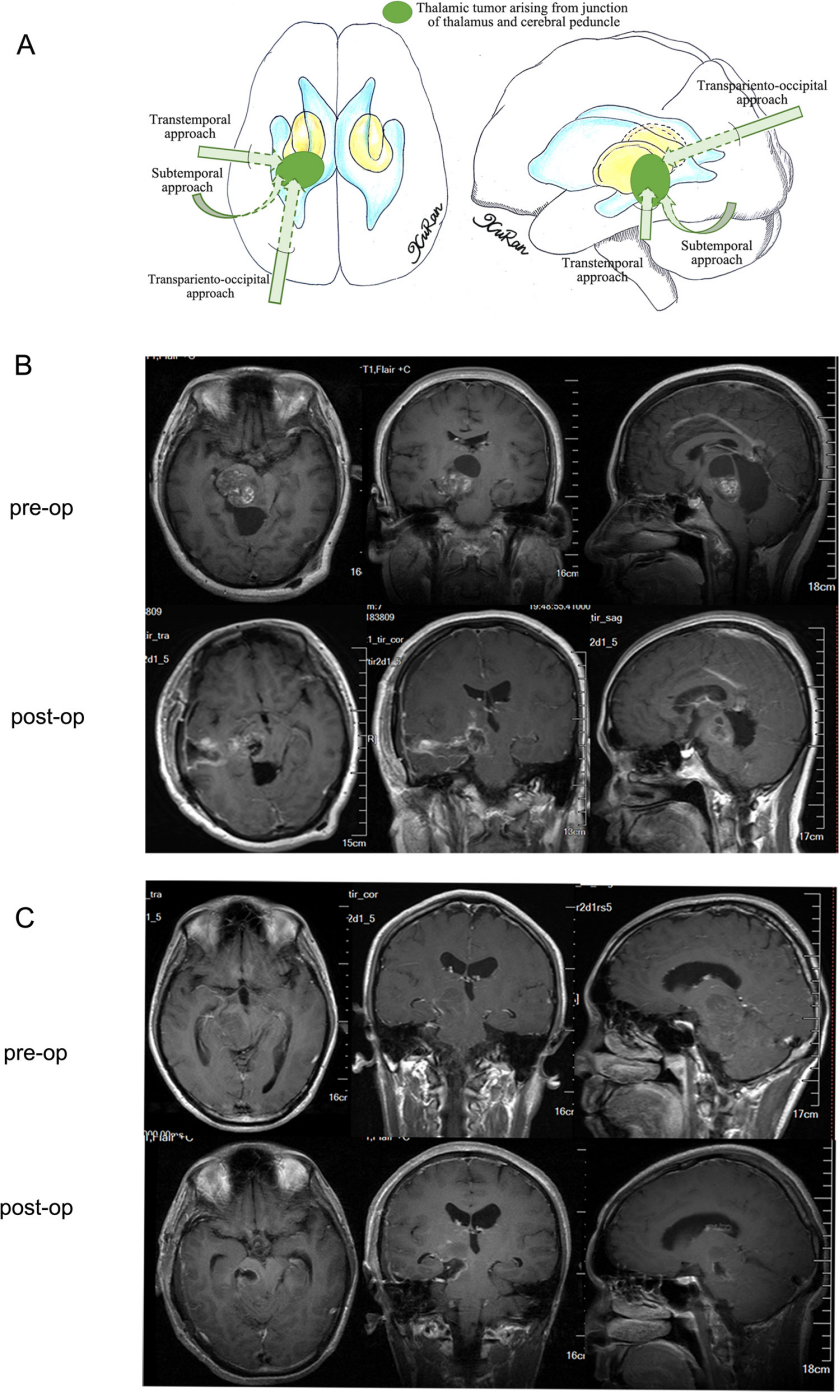
**a** Different surgical approaches to thalamic tumors arising from junction of pulvinar thalamus and cerebral peduncle were showed in the diagram. **b** An astrocytoma was removed subtotally via a transtemporal approach. The tumor arose from the junction of thalamus and cerebral peduncle and extended to both sides equally, and both parts were well exposed via the transtemporal approach. **c** The inferior part of another astrocytoma was well exposed and removed via a subtemporal approach. The postoperative MR image shows that some residual tumor was still present in the thalamic region

For tumors arising from the junction of the thalamus and cerebral peduncle with most of their mass located in the thalamic region, the transparieto-occipital approach was used to remove the thalamic part of the tumor. The inferior part that slightly extended to the cerebral peduncle was also exposed and removed. If the tumor occupied the cisterna ambiens and extended inferiorly to the infratentorial area, the tumor could be exposed by elevating the temporal lobe and making a tentorial incision via a subtemporal approach. If the tumor extended to the thalamic and peduncle to a similar extent, the transtemporal approach was a good choice for both regions. Overall, the transparieto-occipital approach was used in 16 cases (14 %), the transtemporal approach was used in 2 cases (2 %), the subtemporal approach was used in 6 cases (6 %) (Fig. 5), and a stereotactic biopsy was performed in 3 cases (3 %).

Based on both the surgical findings and post-operative MRI findings, complete tumor resection was achieved in 29 cases (26.1 %), subtotal resection was achieved in 54 cases (48.6 %), and partial resection was achieved in 21 cases (18.9 %). In Table 2, data regarding tumor location and the extent of resection are summarized. Taken together, these results indicate that it was difficult to achieve total and subtotal resection when tumor infiltration reached the midbrain.

**Table 2:** Extent of resection based on the anatomic location of the 111 adult unilateral thalamic tumors

Epicenter	No. cases *n* (%)	Extent of resection				Median KPS score (range)	
		Total	Subtotal	Partial	Biopsy	Pre-op	Post-op^a^
Anterior thalamus	20 (18 %)	6	11	2	1	80 (30–100)	90 (50–100)
Lateral thalamus	7 (6.3 %)	2	4	1	0	50 (30–80)	50 (30–90)
Medial thalamus	18 (16.2 %)	4	10	3	1	80 (40–100)	90 (50–100)
Pulvinar	39 (35.1 %)	12	18	7	2	70 (30–90)	80 (0–100)
Junction of thalamus and cerebral peduncle	27 (24.3 %)	5	10	8	3	60 (30–80)	70 (0–90)

*KPS* Karnofsky Performance Scale, *Pre-op* pre-operation, *Post-op* post-operation

^a^Evaluated in three months postoperatively

### Perioperative morbidity and mortality

Five patients died during the perioperative period, two as a result of coma and three as a result of cerebral swelling and infarction. Table 3 summarizes the postoperative clinical features of the other 106 patients upon discharge. For sensory function, visual ability, and ICP, 80/106 cases (84.9 %), 100/106 cases (94.4 %), and 100/106 cases (94.4 %) showed no decrease or improvement in these functional categories, respectively. Regarding motor deficits, only patients with preoperative motor deficits (*n* = 44) could be evaluated for improvement in motor deficits, yet all 106 patients could be evaluated for deterioration of motor deficits. Therefore, preoperative motor deficits were found to improve following surgery in 15/44 cases, while motor deficits deteriorated in 23/106 cases (Table 3). For the latter, myodynamic muscle strength was also found to slightly recover in 15 of these cases within 3 months. A significant functional decrease in patients with deteriorated motor deficits was observed when the intra-operative electrophysiological monitor was used (18 cases in non-electrophysiological stage vs. 5 cases in electrophysiological stage). In addition, new sensory deficits were detected in 16 cases (13 cases in non-electrophysiological stage vs. 3 cases in electrophysiological stage), while a significant increase in ICP was found in 6 cases (these patients underwent cranial decompression), and hemianopia developed in 6 cases. Other complications included speech disorders, memory disorders, involuntary movements, spasticity, seizures, and behavioral problems, which were observed individually or in combination in 16 cases.

**Table 3:** Postoperative clinical features of 106 adults with unilateral thalamic tumors

Clinical feature^a^	Change of myodynamic muscle strength level	No. of patients (%)
Motor		
Improved		15 (14.2)
	1 grade	5 (4.7)
	2 grades	3 (2.8)
No change		68 (64.1)
Deteriorate		23 (21.7)
	1 grade	7 (6.6)
	2 grades	8 (7.5)
	3 grades	5 (4.7)
	4 grades	3 (2.8)
Sensory		
Improved		17 (16)
No change		73 (68.9)
Deteriorate		16 (15.1)
Visual function		
Improved		25 (23.6)
No change		75 (70.8)
Deteriorate		6 (5.7)
ICP		
Improved		60 (56.6)
No change		40 (37.7)
Deteriorate		6 (5.7)
Others^b^		
Improved		30 (28.3)
No change		60 (56.6)
Deteriorate		16 (15.1)

^a^Compared to preoperative clinical features, when the patients were discharged

^b^Other symptoms included speech disorder, involuntary movement, spasticity, seizures, and behavioral problems. Lines in bold indicate total number of patients in that group

Perioperative hydrocephalus was encountered before and after surgical resection. In 42 cases where hydrocephalus was identified preoperatively, four patients underwent ventriculo-peritoneal (VP) shunt placement surgery at another hospital for treatment of severe ICP symptoms. In 28 of the remaining 38 cases, the hydrocephalus improved without VP shunt placement after tumor resection. In the other 10 cases, the hydrocephalus did not improve and eventually a VP shunt was required. After tumor resection, ventricle enlargement was observed in six patients that did not exhibit preoperative hydrocephalus, including five patients who required a VP shunt and one patient who underwent placement of an external ventricular drain.

### Histopathological findings, adjuvant treatment, and survival analysis

Histopathology confirmed the following tumor types in the present series: 88 astrocytomas, 5 oligodendrogliomas, 12 oligoastrocytomas, 3 ependymomas, 2 gangliogliomas, and 1 primary neuroectodermal tumor (Table 4). Of these tumors, 50 were high-grade and 61 were low-grade. All patients with high-grade tumors or progressive low-grade tumors completed postoperative radiotherapy and/or chemotherapy, except for five patients who died during the perioperative period. In addition, 20 patients were lost to follow-up. Of the remaining 86 patients, 40 had high-grade tumors and 44 had low-grade tumors.

**Table 4:** Histological classification of adult unilateral thalamic gliomas of the present cohort (*n* = 111)

Histological type	No. of cases (%)
Astrocytoma	88 (80 %)
low-grade	46
anaplastic astrocytoma	17
glioblastoma	23
Oligodendroglioma	5 (5 %)
low-grade	1
anaplastic	4
Oligoastrocytoma	12 (11 %)
low-grade	8
anaplastic	4
Ependymoma	3 (3 %)
low-grade	2
anaplastic	1
PNET^a^	1 (1 %)
Ganglioglioma	2 (2 %)

^a^ *PNET* primary neuroectodermal tumor

Only 18 patients were alive at the time of the last follow-up (mean follow-up duration, 37.3 months; range, 6–98 months). The median survival times for patients with low- and high-grade tumors were 40 and 12 months, respectively. The 1- and 3-year survival rates of patients with low-grade tumors were 94.7 % ± 3.6 % and 57.7 % ± 8.1 %, respectively, which were higher than those of patients with high-grade tumors (43.2 % ± 7.5 % and 6.8 % ± 3.8 %, respectively; *P* < 0.001). The median survival period was longer for patients who underwent total or subtotal resection compared with those who only underwent partial resection or biopsy (28 vs. 12 months, respectively). The median survival period of patients with high-grade tumors who underwent total/subtotal resection was 5.5 months longer than for patients who only underwent partial resection or biopsy and adjuvant therapy (14.5 vs. 9 months, respectively). The median survival period of patients with low-grade tumors who underwent total/subtotal resection was 12 months longer than for patients who only underwent partial resection or biopsy (12 vs. 30 months, respectively) (Table 5). Compared with previously published series, the survival rate of the present series of adult thalamic tumor patients was lower than that of pediatric patients with either high-grade or low-grade tumors, yet was higher than that reported by Kelly et al. for an adult cohort with high-grade tumors (Table 6). However, the number of poor outcomes indicate that thalamic tumors in adults need to be further studied.

**Table 5:** Comparison of the present series with other published series of thalamic tumors

Series	No. pediatric patients	No. adult patients	WHO grading		Total/subtotal resection	Postoperative improvement in preexisting motor deficits	Perioperative mortality	Median survival rate (months)		5-year overall survival	
			High- grade tumors	Low- grade tumors	% (n)	% (n)	% (n)	High-grade tumors	Low-grade tumors	High-grade tumors	Low-grade tumors
Present series	0	111	50	61	73.9 (82)	34.1 (15/44)	4.5 (5)	12	40	/	/
Cuccia, [2]	26	0	17	9	35 (9)	20 (2/10)	8 (2)	12^†^	34^†^	/	/
Albright AL, [5]	19	0	12	7	84 (16)	/	5 (1)	/	/	/	/
Ozek, [7]	18	0	5	13	89 (16)	/	0	/	/	/	/
Puget S, [10]	54	0	22	32	46.3 (25)	/	4 (3)	21	>60	45.5 %	87.5 %
Kramm CM, [13]	99	0	99	0	19.2 (19)	/	/	/	/	10.8 %	/
Sai Kiran NA, [15]	26	15	22	19	63.4 (41)	32.3 (10/31)	0	/	/	/	/
Steiger, [19]	5	9	10	4	71.4 (10)	/	0	15	21	/	/
Baroncini M, [21]	16	0	9	7	68.8 (11)	/	0	11	37	/	/
Kelly, [23]	15	57	40	32	36 (26)	27 (12/45)	6.9 (5)^‡^	5	41	/	/
Bilgniner B, [24]	45^a^	0	14	31	60 (27)	/	/	15^†^	85^†^	/	/

^a^ implied for patients 3–20 years old

^b^ implied mean survival rate

^c^ implied to be the number of patients that died within 7 days following operation

**Table 6:** The indications and complications of different surgical approaches to thalamic tumors

Surgical approach	Indications	Complications
Anterior transcallosal approach	① The epicenter was located in the anterior thalamus with/without anterior extension to the fontal horn of the lateral ventricle or callosum;	The approach is limited laterally by stretching of the pericallosal artery; some cases suffered from transient mental disorders or memory deficits
	② The epicenter was located in the medial thalamic region with/without extension into the third ventricle	
Transfrontal approach	The epicenter was located in the anterior thalamus and extended too much laterally (over 2 centimeters from the lateral broader to the middle line)	High risk of postoperative seizures
Transtemporal approach	① The epicenter of the tumor was located in the lateral thalamic region with/without lateral extension to the basal ganglia, adjacent lobes, or the gyrus, or extending beneath the temporal cortex;	Visual field defects due to injury to the optic radiation and language disturbances on the dominant side
	② Tumors arising from the junction of the thalamus and the cerebral peduncle and extended to the thalamic and peduncle to a similar extent	
Transinsular approach	The epicenter of the tumor was located in the lateral thalamic region with lateral extension to the basal ganglia	High risk in internal capsule injuries
Subtemporal approach	① Tumors located in the pulvinar with posterior extension toward adjacent structures;	High risk in cortical draining veins and temporal lobe injuries when elevating the temporal lobe
	② Tumors arising from the junction of the thalamus and the cerebral peduncle with most of their mass located in the thalamic region	
Transpariento-occipital approach	Tumors arising from the junction of the thalamus and the cerebral peduncle occupied the cisterna ambiens and extended inferiorly to the infratentorial area	Visual field and memory deficits, due to injury to adjacent optic radiation of the crus fornicis covering the thalamus
Posterior transcallosal approach	Pulvinar tumors that are primarily located medially (within 1 centimeter from the lateral broader to the middle line)	High risk in optic radiation injuries

Multivariate Cox regression analysis was performed to identify variables that independently affected survival (e.g., extent of tumor resection, tumor volume, age, duration of symptoms, preoperative KPS score, histological findings). The results showed that the extent of tumor resection (total/subtotal resection compared with partial resection/biopsy, *p* = 0.005), preoperative KPS score (KPS score ≥ 70 compared with < 70, *p* = 0.003) and histological results (high-grade vs. low-grade tumors, *p* = 0.01) were correlated with patient survival.

## Discussion

A total of 111 operated cases of adult unilateral thalamic tumors were reviewed. Similar to the symptoms described for children affected by thalamic tumors, such adult patients usually present with increased ICP and motor deficits [5, 10]. The tumor often has a mass effect and can lead to hydrocephalus when located in the medial or posterior thalamus, thereby resulting in greater ICP. When the tumor is located in the anterior or lateral thalamus, motor and sensory deficits are usually observed. In contrast, other symptoms, such as seizures, hemianopia, speech disorders, and tremors, are uncommon [6].

Various surgical approaches have been described for the resection of thalamic tumors, including transfrontal, transcallosal, transtemporal, subtemporal, and transparieto-occipital [7, 10, 15]. However, the indications for these approaches have not been clearly described. In the present series, the surgical approach was determined according to tumor location (summarized in Fig. 4 and Table 6). The anterior transcallosal approach was used for anteriorly located thalamic tumors that extended toward the lateral ventricle. However, access through the transcallosal approach is limited laterally by stretching of the pericallosal artery. If the tumor extended too laterally, then the transfrontal approach was preferred, although the latter approach is associated with a high risk of postoperative seizures. Moreover, if the ventricle was not enlarged, then access to the ventricle was difficult without the help of neuronavigation or intraoperative ultrasonography.

If the tumor extends beneath the temporal cortex, a trans-temporal middle gyrus approach is a better choice. A cortical incision between T1 and T2 is recommended to reduce the risk of vessel injury. The risk of internal capsule injuries is lower than with the transinsular approach. For lateral thalamic tumors that extend beneath the insular cortex, a pterional transsylvian transinsular approach seems to be the best option [16]. However, the success of this approach depends on the experience of the neurosurgeon, but yet is also associated with a high risk of internal capsule injuries.

In the case of thalamic tumors that extended medially to the third ventricle, a transcallosal approach was often chosen. If the tumor extended beneath the floor of the lateral ventricle, the floor (top of thalamus) can be incised to access the tumor directly without dissection of the crus fornicis. If the tumor did not reach the lateral ventricle floor, then the third ventricle was accessed via a natural fissure using a transcallosal interfomiceal-transforaminal approach, which allowed cortical incision and crus fornicis injuries to be avoided. Moreover, memory deficits were not found to correlate with this surgical approach, consistent with the results of a previous report [17].

The posterior-interhemispheric-transcallosal approach has been proposed for pulvinar tumors that are primarily located medially [16]. However, this approach cannot be used for laterally extended tumors on account of callosum stretching. For pulvinar tumors that extended posteriorly, the transparieto-occipital transventricular approach was used. However, this approach is associated with visual field and memory deficits due to injury to adjacent optic radiation of the crus fornicis covering the thalamus.

For tumors arising from the junction of the thalamus and cerebral peduncle that are mostly located in the thalamic region, use of a transparieto-occipital approach to remove the thalamic part of the tumor is advocated. Afterwards, the inferior part that slightly extends to the cerebral peduncle can be exposed and subsequently removed. If the tumor occupies the cisterna ambiens and extends inferiorly to the infratentorial area, the tumor can be exposed by elevating the temporal lobe and making a tentorial incision via a subtemporal approach. If the thalamic part is almost equal to the infratentorial part, a transtemporal approach is appropriate for exposure of both parts.

In our group, the extent of total and subtotal resection was less when the tumor infiltrated the cerebral peduncles. Sometimes, it may be impossible to determine the tumor margin adjacent to the cerebral peduncle. Frameless stereotactic guidance or intraoperative ultrasonography guidance was helpful to evaluate the margins in this series. However, the risk of peduncle injury remained high, as even with these techniques, errors in distance calculation are possible. In our group, two patients were comatose and died postoperatively due to intraoperative peduncle injury. Therefore, for cases in which it is difficult to intraoperatively resect the tumor totally or subtotally, partial resection or biopsy may be a better choice, in agreement with a previous report [10].

Intraoperative neuronavigation or ultrasonography guidance is very important for the surgical treatment of thalamic tumors, as these techniques provide the shortest route to the tumor, sufficiently depict the tumor margins, and allow for a postresection assessment of any obvious residue. Diffusion tensor imaging can be used to reveal the corticospinal tract and to determine how the tumor can be resected maximally without inducing corticospinal tract injury [18]. A disadvantage of neuronavigation is that it results in tissue shift after resection or cerebrospinal fluid (CSF) release. However, using ultrasonography guidance, the tumor margin can be monitored intraoperatively without tissue shift and residues can be assessed after resection.

In the present series, five patients died during the perioperative period, all of whom had high-grade tumors. Two of the five patients were comatose with cerebral peduncle injuries. Three of the five patients died as a result of uncontrolled cerebral swelling followed by disruption of blood supply and infarct formation. This mortality rate was higher than that for series with smaller sample sizes (e.g., < 40 patients), but was consistent with, or better than, previously reported series with larger sample sizes (e.g., > 40 patients) (Table 5). Motor, sensory, and visual deficits were observed in cases where the tumors had compressed adjacent structures, especially the posterior limb of the internal capsule. After decompression, function was found to improve. Motor and sensory deficits were the most common morbidities. About half of the patients had transient hemiparesis, which may have been caused by postoperative cerebral swelling that influenced the posterior limbs of the internal capsule. Sensory deficits were due to central thalamic radiation injuries and visual field deficits were caused by visual radiation. Aphasic problems can be caused by pulvinar injuries, as the pulvinar is the site of neuronal projection to the language centers [19]. The high rate of postoperative sensorimotor and visual deficits may be due to the cerebral swelling or heat injury caused by bipolar coagulation. Moreover, the unavailability of intraoperative electrophysiology in the early stage was also another factor impacting postoperative functional deficits.

Cerebral swelling was one reason for the postoperative increase in ICP, which can usually be reversed using medical therapy or cranial decompression. Another cause of increased ICP was postoperative hydrocephalus. In this series, hydrocephalus was treated by tumor resection in 74 % of patients, as CSF drainage can be re-established after tumor resection. In six patients, the CSF pathway was still obstructed postoperatively as a result of cerebral swelling or gliosis surrounding the aqueduct or interventricular foramen of Monro, requiring CSF diversion. Therefore, in our experience, preoperative shunting of thalamic tumors are not necessary due to the potential for neurological degradation or subsequent shunt blockade. Similar findings were published by Gole [20].

The outcomes of the thalamic tumors examined in this series were found to correlate with the histopathological results and the median survival period for patients with low-grade tumors was longer than that for patients with high-grade tumors, consistent with previous reports [2, 5, 7, 21]. The overall survival of low-grade tumors in adults is reportedly lower than that in children, perhaps due to the lower degree of neuroplasticity in adults compared to children [21]. For patients with high-grade tumors, the overall survival period was 12 months, similar to that observed for children. Patients who underwent total or subtotal resection also achieved a better survival rate than those who underwent partial resection or biopsy. This finding is consistent with the results of other studies, which found that maximal safe resection of thalamic tumors is feasible and generally results in a better outcome [10, 19, 22, 23]. These findings support a surgical approach for management of these tumors and imply that extended tumor resection can improve prognosis.

## Conclusions

Deep-seated thalamic tumors, especially in adults, are a challenge for many neurosurgeons. Based on the findings from the present series, surgical removal appears to be an appropriate treatment for thalamic tumors in adults. Moreover, the appropriate surgical approach should be determined based on the location of the tumor and its relationship with surrounding structures. The extent of tumor resection and histological classification are also two very important factors to consider when determining the prognosis of patients with thalamic tumors. Nevertheless, further studies will be necessary to improve the poor outcomes of adults with thalamic tumors.
